# Enabling Remote Responder Bio-Signal Monitoring in a Cooperative Human–Robot Architecture for Search and Rescue

**DOI:** 10.3390/s23010049

**Published:** 2022-12-21

**Authors:** Pablo Vera-Ortega, Ricardo Vázquez-Martín, J. J. Fernandez-Lozano, Alfonso García-Cerezo, Anthony Mandow

**Affiliations:** Robotics and Mechatronics Lab, Institute for Mechatronics Engineering & Cyber-physical Systems, Universidad de Málaga, 29071 Málaga, Spain

**Keywords:** real-time monitoring, bio-signals, remote sensors, cloud robotics, 5G communications, search and rescue

## Abstract

The roles of emergency responders are challenging and often physically demanding, so it is essential that their duties are performed safely and effectively. In this article, we address real-time bio-signal sensor monitoring for responders in disaster scenarios. In particular, we propose the integration of a set of health monitoring sensors suitable for detecting stress, anxiety and physical fatigue in an Internet of Cooperative Agents architecture for search and rescue (SAR) missions (SAR-IoCA), which allows remote control and communication between human and robotic agents and the mission control center. With this purpose, we performed proof-of-concept experiments with a bio-signal sensor suite worn by firefighters in two high-fidelity SAR exercises. Moreover, we conducted a survey, distributed to end-users through the Fire Brigade consortium of the Provincial Council of Málaga, in order to analyze the firefighters’ opinion about biological signals monitoring while on duty. As a result of this methodology, we propose a wearable sensor suite design with the aim of providing some easy-to-wear integrated-sensor garments, which are suitable for emergency worker activity. The article offers discussion of user acceptance, performance results and learned lessons.

## 1. Introduction

Fitness-For-Duty (FFD) refers to the physical and mental ability of a worker to safely perform the essential functions of his or her job [1]. The roles of emergency services are challenging and often physically demanding because they need to adapt to the extreme conditions of disaster environments. Readiness to meet these challenges and demands is a fundamental requirement for staff to work safely and effectively [2]. First responder is a substantial term that includes emergency workers, such as firefighters, police officers and paramedics [3]. For these workers, efficiency in their tasks is decisive because it has a direct impact on time employed on victim recovery and assistance.

In this article, we focus on first responders’ health while on duty, which involves both physical fatigue and psychological aspects such as stress and anxiety. Stress is an immediate response of the body to stimuli that disturb the mental or physical equilibrium of a person [4]. Continuous exposure to stress can cause anxiety and hinder concentration on assigned tasks [5]. Moreover, not only mental but also physical fatigue can provoke an immediate increase in the risk of committing errors and causing accidents [6]. In the long-term, stress has been linked to the three leading physical causes of death in the general population: heart disease, stroke, and cancer [7]. Two of these, heart disease and cancer, are prevalent causes of mortality in firefighters [8].

The resilience and cognitive readiness of emergency workers can benefit from increasing research interest in wearable sensors for biological signal monitoring and analysis. The availability of inexpensive and small sensors for real-time monitoring and connectivity technologies has enabled recent applications such as healthcare [9,10,11], human–machine interfaces [12] and monitoring of workers [13] or elderly people [14].

In previous works, we presented a mission-oriented Internet of Cooperative Agents architecture for search and rescue (SAR) missions (SAR-IoCA) [15] that integrates robotic and human agents in the field with the mission control center. SAR-IoCA allows remote control and communication [16], integrating hybrid sensor networks [17], victim close detection [18] and requests from rescuers [19]. In this work, our goal is to enable remote first responder bio-signal monitoring during SAR interventions. For this purpose, the employed methodology starts with the definition of a set of sensors to measure the main signals of the responder in order to monitor the most interesting aspects of their health. This sensor suite is integrated into SAR-IoCA and used in several realistic experiments. The experience of these experiments in disaster scenarios with actual first responders demonstrates the feasibility of real-time monitoring in extreme conditions while on duty. As a result, a survey to the target community was conducted to obtain useful information to finally propose a design of a wearable sensor suite for bio-signal monitoring. The main contributions of this article are:

- We performed proof-of-concept experiments with a bio-signal sensor suite worn by firefighters in two high-fidelity simulated SAR disaster scenarios. This sensor suite is integrated into a cloud robotics architecture for real-time bio-signal monitoring and data transmission to a Forward Control Center (FCC) via 5G for expert mission supervision.
- A survey was conducted among Spanish firefighters with the aim of collecting their opinion and interest in bio-signal monitoring while on duty.
- Based on learned lessons from these experiments and survey results, we propose a sensor suite with a non-invasive and easy-to-wear design that has reduced interference with emergency worker activities.

This article is structured as follows: after this introduction, Section 2 reviews related work. Section 3 presents the objectives and methodology. Section 4 defines measured biological signals, their features and sensor arrangement on the first responder’s body. Section 5 describes the integration of these sensors in the SAR-IoCA architecture for real-time bio-signal monitoring. Section 6 describes the proof-of-concept in two experiments in high-profile SAR missions. Section 7 analyzes firefighters’ survey results. In Section 8, we propose a wearable sensor suite. Finally, Section 9 offers conclusions and ideas for future work.

## 2. Related Work

Many studies on assessments of common daily-life activities and some types of on-duty workers can be found in the literature with the aim of preventing situations of stress, anxiety or fatigue. In [20], an assessment carried out to detect fatigue in car drivers and aircraft pilots reviewed non-invasive neurophysiological measures, such as electroencephalograms (EEG), electrocardiograms (ECG), electrooculograms (EOG) and eye movements and concluded that a single method for fatigue evaluation is not sufficient. Bio-signals were also recommended for improving the ergonomics of heavy equipment operation and worker’s health [21], where EEG is not feasible due to electromagnetic noise inside heavy machinery, being the most informative bio-signals ECG and galvanic skin response (GSR) data. Furthermore, studies in nuclear power plant workers were conducted in [22] for monitoring psychological distress using EEG at certain brain areas where reduced alpha and increased beta activity in cortical areas are associated with anxiety, but conclusions were limited by the small sample size and the difference of EEG signals for each person. In [23], an evaluation was performed to develop a concept for the assessment of fatigue, acute stress and combat/cognitive readiness in military battle tank crews by recording the ECG and the physical activity and eye movements during sleep, but no experiments were shown. Studies were also out to detect intimate partner violence [24], where monitoring of heart rate (HR), pulse, body temperature, electrodermal activity (EDA) and the brain’s electrical activity was proposed for training machine learning algorithms to generate automatic aggression alarms without the victim’s conscious action, but no experimental validation is available.

In the field of biological signal analysis, solutions for some specific tasks have been proposed by processing and extracting useful features from bio-signal data. Some applications have been human–machine interfaces, such as robot control using three electromyography (EMG) signals from a human arm [25] or hand gesture recognition also using EMG [26]. In the development of new human–machine interfaces using non-invasive methods to decode a user’s intentions, a review of sensor fusion methods can be found in [12]. The review concluded that the fusion of two or more myography methods leads to better performance. Moreover, near-fall detection for the elderly and people with Parkinson’s disease using EEG and EMG [27] and machine learning based on stroke disease prediction using ECG and photoplethysmography (PPG) [28] are examples of applications in medical settings. Another specific application is real-time emotion classification, where [29] proposed a convolutional autoencoder based on PPG and GSR signals from experiments where subjects watched a short video and marked self-assessment labeling of two or three classes (positive, negative or neutral emotions). Similarly, Ref. [30] addressed emotion classification using ECG, PPG, a respiratory belt and a thermal infrared camera to register facial skin temperature to train a feedforward neural network for four classes.

This interest in the analysis of biological signals requires the existence of datasets and benchmarks, where the research community may find a large amount of data to train their machine learning algorithms and propose new methods. AffectiveROAD [31] included physiological and environmental sensors for driver attention assessment. In [32], biometric data were collected from nurses in a hospital during the COVID-19 outbreak, using a wristband while on duty. WESAD [33] is a multimodal dataset where subjects were exposed to stressful activities, even if it is limited by low sample size and lack of diversity.

Some works have focused on firefighters and emergency responders. Thus, the firefighter assessment of stress test (FAST) [8] proposed a behavioral–analytic model for situational analysis through tests and cognitive interviews to identify stressful scenarios, but the main evaluated factors were related to office tasks, such as responding to calls, administrative stress and overwork. In the field, work with firefighters has been carried out to propose smart helmets as wearable devices for emergency issues or potential harm by monitoring bio-signals. A human-centered design of a smart helmet named FireWorks [34] was proposed, collecting user needs by interviewing 80 firefighters. FireWorks includes an array of sensors to collect body temperature, HR and motions and is connected to a smartphone through Bluetooth Low Energy (BLE) to collect data locally and to synchronize with a cloud database using a radio link. A similar design was proposed in [35] using a LoRa (Long-Range Radio) wireless protocol to achieve long-range and low-power communication with the cloud database. Both designs were proposed to alert the supervisor of potential health or emergency issues, such as dehydration, potential falls or abnormal heart rates. We are interested in monitoring an extended set of bio-signals that is not limited to the head, including other body parts, such as the forearm for EDA and the chest for ECG and breathing. For this purpose, integrated platforms, such as Multimed [36], are interesting. Multimed offers hardware and open-source control and processes software for real-time bio-signal monitoring. However, this platform is designed for laboratories and is not appropriate for wearable devices for on-duty workers. Commercial products can be found for military [37] or first-response [38] but with a limited set of sensors.

Therefore, there is an increasing interest in biological signal monitoring and analysis, where a combined set of sensors is needed to study situations of stress, fatigue or anxiety. However, there is a significant gap in the development of real-time biosensor systems and experimentation in a huge variety of domains.

## 3. Objectives and Methodology

### 3.1. Objectives

The objective our this study is enabling remote first responder bio-signal monitoring during realistic SAR missions, integrating a set of biosensors in the first responders’ body without disturbing their duty. We note the following milestones:

- Selecting a set of biological sensors to monitor relevant first responder bio-signals, useful for subsequent analysis to detect stress, anxiety or fatigue.
- Proof-of-concept experiments for remote bio-signal monitoring in SAR-IoCA architecture in SAR missions during first responder interventions. In order to achieve continuity in bio-signal data, two schemes are considered: real-time communication and local data storage to prevent data lost during transmission to the mission control center.
- Design proposals based on the acquired experience in experiments to improve the performance in future exercises.

### 3.2. Methodology

In order to meet challenges on first responder health monitoring while on duty during realistic SAR missions, this problem is addressed in the following steps:

1. A sensor suite is proposed to measure the appropriate biological data that meet the requirements of monitoring the most important factors in first responders’ health, such as stress, fatigue and anxiety.
2. Once selected, the sensor suite is integrated into a cloud robotics architecture for remote control and communication between agents in the field (human and robotic) and the mission control center.
3. Some experiments in high-impact SAR exercises in simulated disaster scenarios are conducted with real first responders during real-time interventions.
4. After the on-field experience in SAR missions on biological signal monitoring, a survey among the end-users is conducted with the aim of obtaining useful information with only a few questions. These questions were designed to know the firefighters’ interest in bio-signal monitoring and their willingness to participate in this line of research.
5. Finally, as a result of the experiences on the field and the survey results, an easy-to-wear design to put on the sensor suite easily is proposed in order to avoid sensor fixing problems and reduce sensor arrangement before first responder interventions.

The following sections describe and illustrate the development and design of a wearable sensor suite for real-time biological sensor monitoring of first responders during interventions in SAR missions.

## 4. Measured Bio-Signals

**Figure 1:**
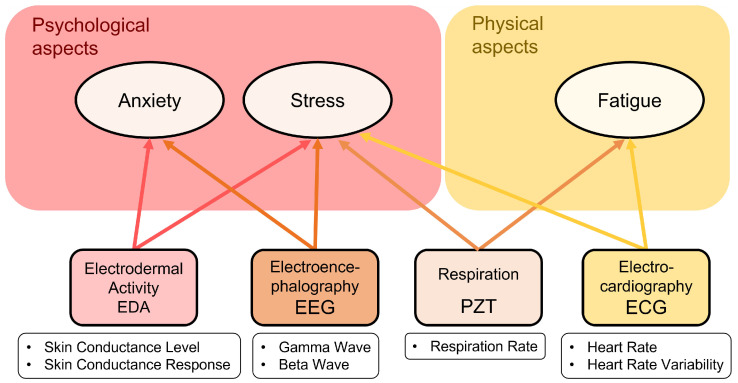
Relation between measured bio-signals and psychophysical aspects.

**Figure 2:**
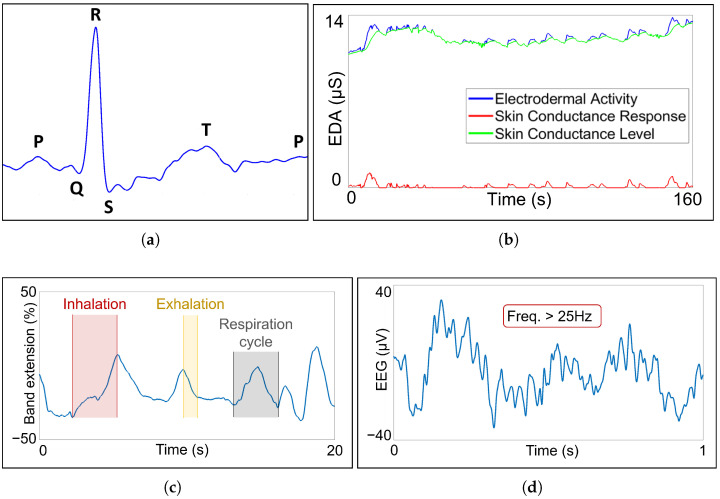
Measured bio-signals: (**a**) ECG with characteristic points: P, Q, R, S and T; (**b**) EDA with skin conductance response and skin conductance level components; (**c**) PZT signal with one inhalation, one exhalation and one complete respiration cycle highlighted; (**d**) gamma wave EEG signal, with a frequency higher than 25 Hz.

According to the objectives defined in Section 3, this work proposes a set of measurable signals that are suitable to monitor and detect both psychological (anxiety and stress) and physical (fatigue) aspects for first responders on duty. In particular, we propose measuring ECG, EDA, respiration data and EEG brain waves, as shown in Figure 1. Examples of measured signals are illustrated in Figure 2.

Regarding psychological aspects, anxiety is related to EEG and EDA signals. EEG measures brain electric activity, which can be indicative of concentration or relaxation states, and EDA values increase with the presence of anxiety [20,22]. Moreover, for stress detection, although the four bio-signals are used, the most important feature is EDA because of its relationship with the sympathetic nervous system (SNS), since this system acts as a trigger for stress. HR and respiration rate increase and EEG gamma waves appear with a stress state [20,22,24,39].

Physical fatigue (referred to just as fatigue) is linked to PZT and ECG signals. ECG measurement allows monitoring the effort during interventions. The respiration (PZT) sensor gives information about breathing, whose rate will tend to increase when physical effort raises [20].

ECG measurement (see Figure 2a) allows knowing HR and its variability (HRV). HR will increase during exercise and will decrease while resting. HRV measures the difference in time between heartbeats.

EDA monitoring gives feedback about sweat gland secretion, which is related to SNS activity. Arousal or relaxation states are translated into sweat production or its absence, respectively. EDA consists of two components, as seen in Figure 2b:

- The skin conductance level (SCL) is a tonic component that changes over time constantly depending on factors such as hydration.
- The skin conductance response (SCR) is a phasic component with short-lasting changes provoked by stimuli.

The respiration rate is obtained from the PZT sensor (see Figure 2c). Hyperventilation or hypoventilation states can be detected thanks to PZT sensor information.

Electric activity of different zones of the brain is recorded by EEG sensors. Each brain zone has different functions, and the recorded signal wave type is dependent on the signal frequency. Beta (12–25 Hz) and gamma (>25 Hz) (see Figure 2d) waves are expected to appear in experiments as representative of active mind and concentration states, respectively. The other three types of EEG waves, alpha (8–12 Hz), theta (4–8 Hz) and delta (0–4 Hz), are representative of restful or sleepy states.

Finally, requirements for a wearable monitoring system are measurement accuracy and reliability, ability to present (high level) real-time readiness information, data transfer reliability, durability and ruggedness, small size and weight, long battery life, fault-tolerance and easiness to wear, use and maintain [23]. It is also important that the system does not cause harm to first responders, nor compromise their performance.

## 5. Integration in a Cloud Robotics Architecture

This section presents the architecture that integrates devices described in the previous section and the particularities of this integration.

### 5.1. SAR-IoCA Overview

In a previous work, an Internet of Cooperative Agents architecture (X-IoCA) was presented [15]. It consists of a cloud robotics scheme that integrates heterogeneous sensor networks and robots, multi-edge computing and fifth generation of mobile network (5G) communications in cooperative field tasks between humans and robots. X-IoCA has two components:

- An Internet of Robotic Things (IoRT).
- A feedback information system (FIS).

The first part of the X-IoCA is the IoRT, composed of sensor networks and entities that carry or wear end-devices, such as sensors or actuators. End-devices belong to two different network types:

- Hybrid heterogeneous wireless transceiver network (H2WTN), which detects sensor events at long-range for obtaining data from sensor-nodes or concentrator-nodes in the area of operation and at short-range for detection of agents, i.e., vehicles, people or animals.
- ROS-based sensor network, which obtains information from integrated sensors on agents, such as acceleration from inertial measurement units (IMU), position from global positioning system (GPS), mapping and localization, recording via video or audio and monitoring of bio-signals.

The other part of X-IoCA is the FIS, consisting of two components for data monitoring and processing:

- X-FIS, which collects data from non-ROS sensors of H2WTN for the purpose of monitoring the operation field and generating points of interest and controlling cooperative robots in response to the received information, thanks to an integrated global path planner.
- ROS-FIS [16], which processes and monitors information of the ROS-based sensor network, with a main computer (MPC) in each edge sharing local area network (LAN) with secondary PCs (SPC).

The IoRT and the FIS are connected through multi-access edge computing (MEC) centers, which can be either cloud or local edges. Cloud edges are remote edges, while local edges correspond to local hosts in the operation area. Both types of edges are distributed between FIS elements, while only local edges are available as IoRT elements. This way, information and data do not share the same physical site, so any agent can use resources that are not physically accessible. All communication is conducted through 5G, which supports high bandwidth, low latency (10–12 ms) [15] and real-time requirements [40]. Moreover, 5G includes features, such as quality of service (QoS) aware applications which may prioritize a part of the traffic [41]. It is even possible to create an ad hoc private 5G network, independent of public operators, to serve a limited geographic area in the event of an emergency [42].

A classification attending to the constituents and their physical location is:

- End-devices at the physical site where data is acquired.
- Fog, composed of cloud and local edges, and switches and 5G customer-premises equipment, which allow sharing data between their respective PCs and hosts.
- Cloud elements, which present no physical location, belonging to independent processing centers: (1) a virtual machine host, (2) a computing center and (3) the master node of the ROS network.

The implementation of X-IoCA for SAR missions is called SAR-IoCA. This architecture presents two MEC centers:

- The Forward Control Center (FCC), which acts as a local edge in the operation area.
- The Base Control Center (BCC), acting as a cloud edge located in a distant lab building and a replica of the FCC, provides redundancy to the system.

Biosensors are integrated into this architecture because of previous and successful applications for robot and human communication and control in disaster scenarios [15,16,17,18,19].

### 5.2. Integration of Bio-Signal Monitoring in SAR-IoCA Architecture

First responder monitoring requires just a part of the SAR-IoCa architecture. H2WTN and SAR-FIS (X-FIS version for SAR missions) are not required in this particular case.

**Figure 3:**
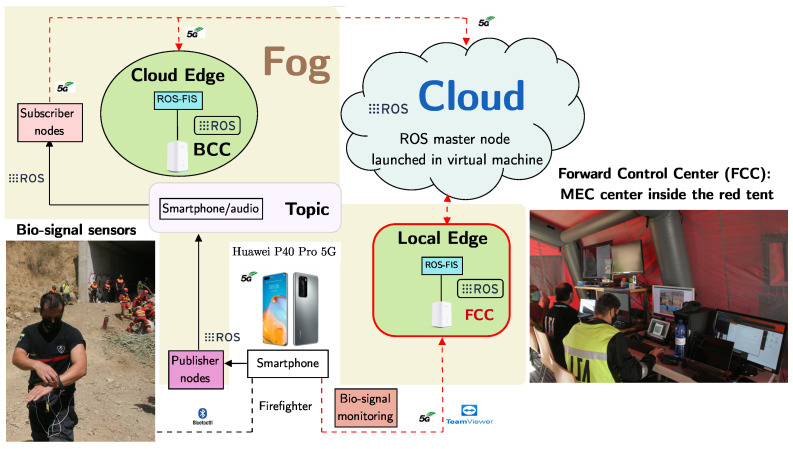
Part of the SAR-IoCA architecture for real-time bio-signal monitoring.

Regarding the part of the SAR-IOCA architecture shown at Figure 3 and the earlier classification of the physical position of X-IoCA elements, it can be distinguished:

- Bio-signal sensors and a smartphone, which records audio, as end-devices.
- The FCC and the BCC inside the Fog.
- The ROS master node launched in a virtual machine, both belonging to the Cloud.

In this particular case, Fog and Cloud elements constitute the ROS-FIS. The smartphone takes part in the ROS-based sensor network, publishing audio recorded from its integrated microphone [19] during a first responder intervention. The ROS master node hosted in the Cloud allows the MPC at the FCC to subscribe to the audio node published by the smartphone, obtaining the audio information. Likewise, the MPC at BCC can subscribe to the audio node too if desired. The smartphone also acts as an intermediate node between the BITalino board, which transmits via Bluetooth bio-signals recorded by physiological sensors on a SAR agent, and the FCC, where real-time bio-signals of the agent are displayed on a screen thanks to the 5G network and remote access software.

**Figure 4:**
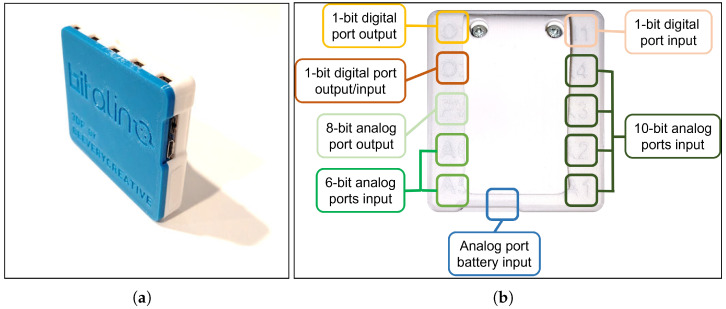
BITalino Core BT board: (**a**) assembled board for measuring bio-signals; (**b**) device ports where sensors and actuators can be connected.

According to the requirements for a wearable monitoring system referred to in Section 4, the device used to measure physiological data from a responder is a BITalino Core BT (see Figure 4), developed by PLUX (Lisbon, Portugal) [43], which consists of a board with:

- Eight analog ports.
- Three digital ports.
- Connection via Bluetooth, with approximately 10 m of range.
- Sampling rate at 1, 10, 100 or 1000 Hz.
- Energy consumption about 65 mA.
- 3.7 V 700 mAh lithium-ion polymer Li-Po battery.

**Figure 5:**
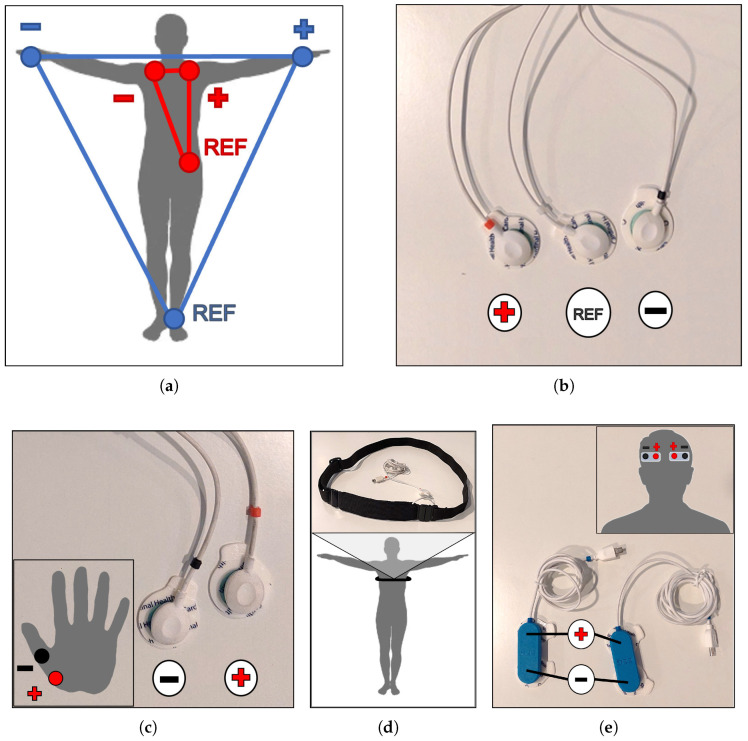
Sensor placement: (**a**) lead I Einthoven configuration; (**b**) ECG electrodes; (**c**) EDA electrodes and their placement on the palm; (**d**) PZT chest-band and its location around the chest; (**e**) EEG electrodes and their location on the forehead.

Bio-signals are measured thanks to pre-gelled self-adhesive disposable Ag/AgCl electrodes shown in Figure 5. Sensors and actuators are connected by cable to the sockets of the board. The BITalino case has a docking clip to attach the device to any garment. BITalino can be linked via Bluetooth to a computer or a smartphone in order to show data collected by sensors thanks to the OpenSignals software suite, also developed by PLUX. This suite allows starting and finishing the recording, showing real-time acquired bio-signals and storing recorded sensor raw data in the internal memory of the device linked to BITalino. Board recorded and transmitted data require a conversion from raw data (10 or 6 bits size) to International System of Units (SI) data using transfer functions provided on sensor datasheets [44,45,46,47].

ECG electrode placement on the responder is based on the lead I Einthoven configuration (see Figure 5a,b), measuring from the right collarbone (negative electrode, sensor black sleeve) to the left collarbone (positive electrode, sensor red sleeve), with the reference electrode (sensor white sleeve) positioned on the left iliac crest. This configuration is the closest placement to the right arm, the left arm and the left foot, respectively, considering the 30 cm long cables used by the ECG sensor.

EDA electrodes were placed on the non-dominant hand of first responders, with the negative electrode (sensor black sleeve) located on the metacarpus of the thumb and the positive one (sensor red sleeve) put on the scaphoid, as shown in Figure 5c. This disposition was recommended by PLUX in the EDA sensor datasheet although electrodes can also be placed on other parts of the body, such as feet, armpits, abdomen or chest. The ergonomics of responders was the major factor in the electrode arrangement decision.

The PZT sensor is integrated into a wearable chest belt and measures displacement variations during respiratory cycles. The chest belt was placed under the chest of the first responders, with the sensor on the front. Upper or lower position was also possible, but volunteers felt more comfortable with the belt in that position (see Figure 5d).

EEG electrodes were disposed on the forehead of the responder, the only place not covered by the helmet (see Figure 5e). This way, the electric activity of the frontal lobe, which controls planning, problem solving, speech, voluntary movements and emotions, was measured.

## 6. Experiments

**Figure 6:**
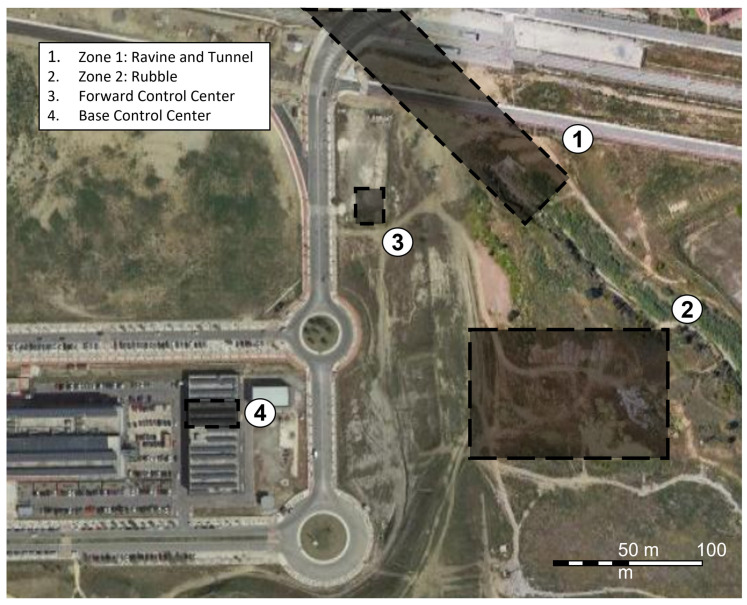
Aerial view [48] of the area of experimentation in new technologies for emergencies (LAENTIEC), where areas indicate relevant locations.

The exercises were conducted as part of an annual workshop organized by the chair of security, emergencies and disasters at Universidad de Málaga (UMA). Exercises were held in two different workshops, on 18 June 2021, and 3 June 2022, respectively. The purpose of the workshops is providing a framework for testing and evaluating new technologies under realistic conditions with the cooperation of members of governmental and non-governmental emergency response organizations. The exercise site was located within the UMA campus, in a 90,000 m${}^{2}$ plot called the area of experimentation in new technologies for emergencies (LAENTIEC). LAENTIEC was divided into several zones (see Figure 6). Although exercises were carried out in two different years, zones remained unchanged:

1. Zone 1, a natural ravine, a creek and a storm drain tunnel.
2. Zone 2, a rubble zone.
3. FCC, located in a tent, where FIS is the interface with vehicles and sensors in the field.
4. BCC, in a laboratory out of the area of operations, being able to share tasks with the FCC to reduce workload.

In previous work using hybrid sensor networks in SAR missions [17], some experiments were conducted in 2018 and 2019 to test the continuity in sensor data transmission in poor coverage conditions. These experiments consisted of data transmission from mobile sensor nodes, allowing local storage to maintain continuity in sensor data and then transferring their local database when the nodes were back to the coverage area. Following this strategy, the 2021 exercise focused on first responder real-time monitoring, while the aim of the 2022 exercise was on local database recording of bio-signals.

### 6.1. Real-Time Monitoring Exercise

**Figure 7:**
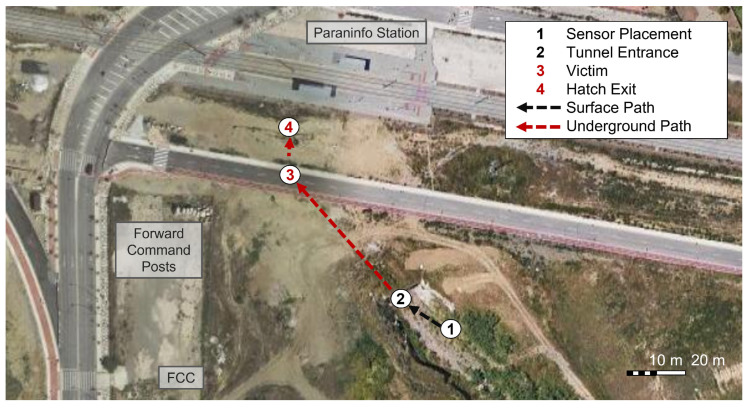
Aerial view [48] of Zone 1. Surface operations in black; underground operations in red.

On the 2021 Workshop, the experiment consisted of a SAR mission in a disaster scenario, which simulated moments after an earthquake that caused a fire with victims trapped inside crushed vehicles into a stormwater drain tunnel (Zone 1 in Figure 6). Figure 7 represents the location of the main milestones in the exercise. Access to the tunnel was possible both from the creek and a hatch located next to Paraninfo metro station (points 2 and 4, respectively). The tunnel has a thickness of about 1 m and a length of about 100 m between the entrance and the hatch and 185 m in total, with a tunnel height of about 7 m and metro tracks above it.

A fireman from the Royal Fire Department of Málaga volunteered for being monitored with ECG, EDA and PZT sensors. BITalino Core BT, sampling at 1000 Hz, was connected to a smartphone, a Huawei P40 Pro 5G (Shenzhen, Guangdong, China), which had the OpenSignals app installed. The smartphone was operated remotely from the FCC thanks to the TeamViewer app, developed by TeamViewer AG (Göppingen, Waden-Württemberg, Germany). This app also allowed data transfer generated by OpenSignals and stored in the phone’s internal memory to a computer. The smartphone was connected to a 5G network to allow communication with the FCC. Audio was transmitted through ROS thanks to the specially designed UMA-ROS-Android app [49] running in background mode. To avoid any kind of undesired interaction with the tactile screen of the phone, the Touch Lock app from Brink Technologies (Veliko Tarnovo, Bulgaria), was used. No instructions were planned to be given from the FCC to the fireman through the cell phone or any other device. Communication from the FCC to the operation area was limited to a phone call to a researcher in charge of the field real-time experiment, if necessary.

**Figure 8:**
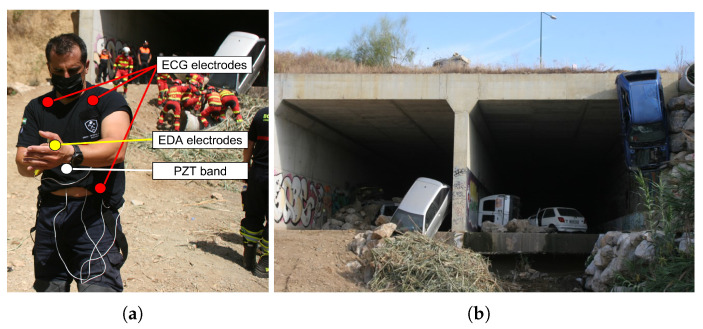
Real-time monitoring exercise: (**a**) deployment of bio-signal measurement devices on first responder in the initial area of operations; (**b**) tunnel entrance. Left side entrance was used.

Before beginning the exercise, at point 1, skin zones where electrodes were going to be placed were cleaned with alcohol. Then, the PZT belt was adjusted to the thorax of the firefighter, and electrodes were stuck on their positions (see Figure 8a). Next, the board was attached to the belt of the fireman, and the smartphone was stored in one of the pockets of his vest. The exercise started at point 2 (see Figure 8b). A group of firemen, including the monitored one, initiated the tunnel exploration searching for potential victims inside.

After 45 min of exercise, firemen exited by the hatch, and the volunteer took off the chest-belt and electrodes, giving sensors, board and smartphone back and proceeding to explain the development of the exercise inside the tunnel: A victim was found at point 3, next to some stairs, which connected with the hatch. The victim needed medical evacuation on a scoop stretcher, so firemen decided to evacuate exiting through the hatch. The space in the stairs was too narrow to let the stretcher move, and such after several attempts, the evacuation was cancelled, concluding the exercise due to accumulated exercises during the day. The fireman also stated that the sensors did not interfere during the exercise except for EDA electrodes which peeled off, so electrodes had to be stuck again.

**Figure 9:**
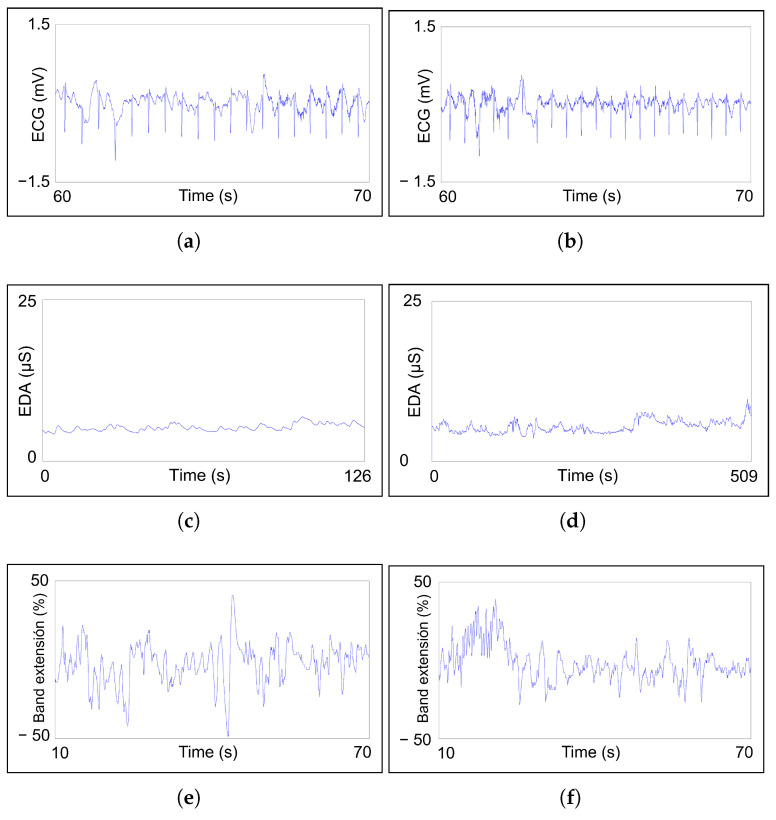
Bio-signals recorded during a real-time monitoring exercise. On the left, recordings corresponding to the first measured interval (126 s). On the right, recordings of the second measured interval (509 s): (**a**,**b**) 10 s sample of ECG recording to show ECG signal intervals; (**c**,**d**) complete EDA recording; (**e**,**f**) one-minute sample of PZT recording.

Despite the duration of the experiment being 45 min, bio-signals could not be recorded during the whole intervention. Real-time bio-signals were monitored for two different intervals of 126 and 509 s corresponding to the initial phase of exploration (see Figure 9). When the OpenSignal app stopped working for the first time, a new bio-signal recording was initialized. Finally, the connection was completely lost because of the interruption of the 5G signal inside the tunnel. Thus, the period where the highest level of stress or fatigue was expected was not recorded.

### 6.2. Local Recording Exercise

**Figure 10:**
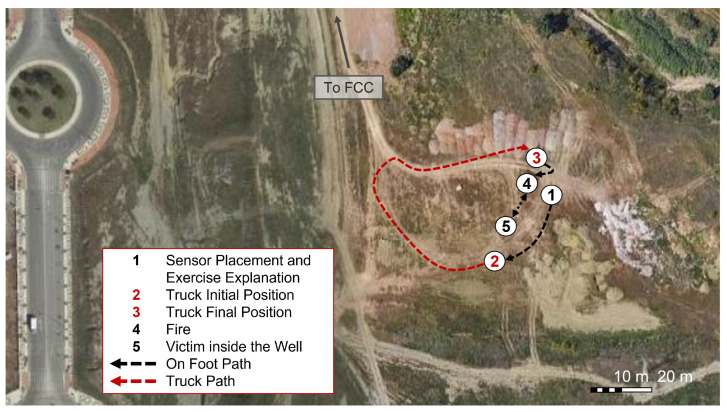
Aerial view [48] of Zone 2. Truck operations in red; on-foot operations in black. Note that fireman moved in both directions between the fire and the victim inside the well.

The second experiment was held on 3 June 2022. Several modifications were implemented regarding last year’s exercise:

1. The exercise took place in zone 2 (see Figure 6), a rubble zone with concrete pipes laid out to simulate, e.g., wells (Figure 10 includes the location of main milestones along the exercise).
2. The volunteer belonged to the Fire Department of Benalmádena, being a different fireman from the previous year.
3. A Samsung S20 FE 5G, by Samsung (Seoul, Republic of Korea) was used instead of the Huawei device.
4. Two EEG sensors were placed on the forehead of the firefighter, in addition to the previously used ECG, EDA and PZT sensors.
5. Electrodes were fixed with tape to the skin of the volunteer for avoiding detachment in the course of the exercise.
6. A wearable 2-megapixel video camera connected to a cloud-based video service delivery platform, BlueEye, manufactured by RedZinc (Dublin, Ireland), was mounted on the helmet of the fireman, with camera connection to the smartphone via a cable with type-C USB connector. This service included accessories, such as the cell phone used in this experiment and its docking clip case.
7. No direct communication between the researcher and the FCC nor phone and the FCC was planned, so the bio-signal recording would be stored in the internal memory of the phone, and video and sensor recording would initialize on site.

Originally, the volunteer was supposed to go into a well (point 5 in Figure 10) to rescue a trapped victim inside. According to the planned exercise, with the aim of interfering the least, the board and the smartphone were attached to the belt at the back of the fireman after skin cleaning and putting ECG, EDA and EEG electrodes and the PZT chest-belt on. The sampling rate of the board was 1000 Hz. However, following instructions from the fire chief, the firefighter participated in a different exercise, which consisted of a fire inside a concrete pipe (point 4) to be suppressed with a cutting extinguisher—a kind of fire hose connected to a water pump on one side and to a hand lance, which mixes water and abrasive for drilling materials, on the other. This new exercise included driving a fire truck and parking next to the water pump. Sensor placement was interrupted several times because of the new instructions received by the fireman, lasting about 15 min.

**Figure 11:**
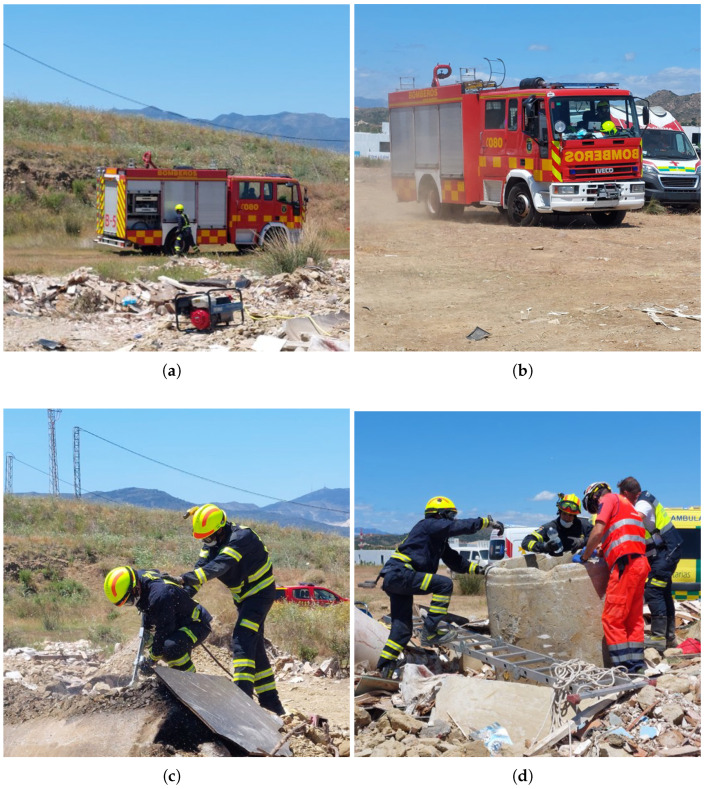
Sequence of the local recording exercise: (**a**) closing truck compartments; (**b**) driving the fire truck; (**c**) using cutting extinguisher; (**d**) Helping at well evacuation.

The exercise started at point 1, with the fire chief explaining the exercise and how to use the cutting extinguisher. After this first part, the firefighter ran alone to the truck (point 2) to store several tools (see Figure 11a). After closing all compartments, the fire truck was driven to point 3, near the water pump and the concrete pipe (see Figure 11b). While seated in the truck, the phone came out of the case. As the fireman got out of the cabin of the truck, the cable disconnected from the smartphone, which fell onto the seat. The image of the video recording froze immediately but audio from inside the cabin continued being recorded. The Bluetooth connection of BITalino was maintained, although when the firefighter started to unload tools from the bottom of the truck, data acquisition began to save wrong values of bio-signals, swinging between threshold values of the sensors, until the distance between the smartphone and the board exceeded Bluetooth range [43] and the transmission ended. The exercise continued with no one having noticed the lack of the smartphone in its case; after several attempts, the water pump was started, and the fire was extinguished by the volunteer (see Figure 11c) from different sides of the pipe (the rescue at the well, see Figure 11d, was in between).

**Figure 12:**
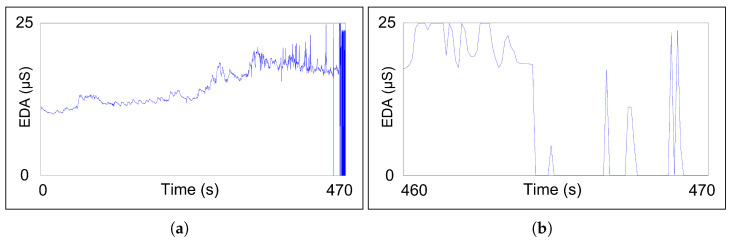
Parts of EDA recorded during local recording exercise: (**a**) sample from the start until the 470th second, showing the difference between the normal recording and the final wrong values; (**b**) beginning of wrong EDA recording at the 464th second. This error occurred in the other four measurements.

Just after finishing the exercise, the fireman approached to give back the cell phone and the board and be interviewed, realizing that the phone had fallen somewhere, being found on the seat of the truck. During the interview, the volunteer stated that sensors had not interfered with his duty, although ECG electrodes had fallen off at the end of the exercise. Even though it was his first use of the cutting extinguisher and it took several attempts to start the water pump, the firefighter stated that he had not felt any kind of stress during the exercise because of his training to deal with dangerous and extreme situations, but he reported that the high working pressure of the cutting extinguisher caused fatigue and stiffness in his arm muscles. In the end, a recording 464 s long (7 min, 44 s) of the exercise was stored (see Figure 12) and documented with several videos and photos.

### 6.3. Evaluation of the Exercises

The experiments carried out were used for measuring and monitoring firefighters during SAR missions developed in open-field realistic exercises. Time required for electrode placement was similar on both exercises, about fifteen minutes (from 12:03 until 12:18 in 2021, and from 12:21 until 12:36 in 2022). Several interruptions happened during these preparations, so this time can be reduced easily in future experiments.

The first exercise allowed us to check that a real-time monitoring of a fireman was possible in a 5G network with a set of physiological sensors (ECG, EDA and PZT) transmitting health information to the FCC. BITalino Core BT was demonstrated to be effective for bio-signal acquisition (not invasive, enough battery life and reliable data transfer to the via-Bluetooth-connected cell phone). By contrast, electrodes did not remain stuck to the skin of the responder in a situation that implies movement and sweating, hindering his labour. As the exercise took place inside the tunnel, the 5G connection was compromised, losing signal soon after the firefighter went into the tunnel. This is a limitation imposed by the trade-off offered by 5G between favorable low latency and data rate against low obstacle penetration [50]. LoRa technology is limited by requirements for public use of a frequency band, which decreases the length of sent packets and the sending frequency in a way that precludes obtaining enough data to represent high-frequency bio-signals, such as ECG. Furthermore, LoRa transmission characteristics are not suitable for monitoring a high number of first responders. BLE and ZigBee technologies have a short range, which limits the direct transmission of bio-signals to the MEC or requires a larger infrastructure compared to the use of a 5G network, adding more complexity [51]. Furthermore, the lack of video recording meant that the only way to know what had happened during the exercise was the subsequent interview with the volunteer. From a 44 min and 22 s exercise, 10 min and 35 s of biosensor signals were recorded, 23.85% of the total time.

The second exercise, during the 2022 Workshop, served for implementing a new strategy aimed at storing recorded data in a low coverage 5G signal scenario instead of real-time monitoring and trying to solve two of the problems that were found the previous year:

- Electrode fastening was improved with adhesive tape.
- A video camera was mounted on the helmet of the fireman to obtain footage from a first-person perspective.

Moreover, two EEG sensors were added to the previous year bio-signal acquisition setup.

The smartphone and the board were attached to the firefighter’s belt to interfere with fire extinguishing tasks as little as possible. However, during the exercise, the firefighter had to drive a fire truck. The placement of the smartphone was not suitable and fell out of its flexible case and stayed in the truck, losing video and Bluetooth connection. While exercise duration was 38 min and 10 s, just 7 min and 44 s of bio-signals were recorded properly, 20.26% of the total time.

From one year to another, the fastening of electrodes improved with adhesive tape as an “easy but effective” solution, and a video recording system was added, although it caused new errors. Design failures detected on the smartphone case and cable connection were reported to RedZinc, improving their performance with a more rigid case and a tighter cable connection to the USB port.

Regarding energy consumption, the BITalino Core BT consumption is about 65 mA with a capacity of 700 mAh, resulting in an autonomy of about 10 h. Moreover, the battery level of the smartphone after the real-time monitoring exercise (44 min duration) was 76% and after the local recording exercise (38 min duration) was 85%.

In general, the experiments provided some insight regarding applicability issues such as electromagnetic interference and energy consumption. As for electromagnetic interference in communications, this was not explicitly considered in the experiments, as the different technologies in the SAR-IoCA architecture used different bands: 3.5 GHz for 5G, 2.4 GHz for Bluetooth and 868 MHz for LoRa. However, it is an important point for future work in case of monitoring several first responders at the same time. The gain of EEG sensors (41,782) was much higher than the gains of the other sensors (e.g., 1100 for ECG, and 1 for PZT) [44,45,46], so EEG is more sensitive to electromagnetic noise and may not be reliable in some environments [21]. In addition to the required improvements regarding electrical shielding, the proposed sensor configuration offers some redundancy for stress and anxiety detection by combining EEG and EDA.

In both exercises, firemen valued as “remarkably positive” the research on real-time monitoring of bio-signals, not just for stress, but for any kind of health glitch which could lead to dangerous circumstances, bearing in mind extreme situations that firefighters have to face in their duty. Both assessed that time to set up electrodes and devices should be reduced as much as possible.

## 7. Firefighter Survey for Bio-Signal Monitoring

A survey was conducted in order to gather firefighters’ opinions on bio-signal monitoring and analysis while on duty. After the on-field experience in realistic scenarios, the aim of this survey was to check the end-user interest in bio-signal monitoring, asking their opinion about the most important aspects of mental and physical health in their duty and which parts of the body should be avoided for placing sensors in order not to hinder their work. Answers were completely voluntary and anonymous.

**Figure 13:**
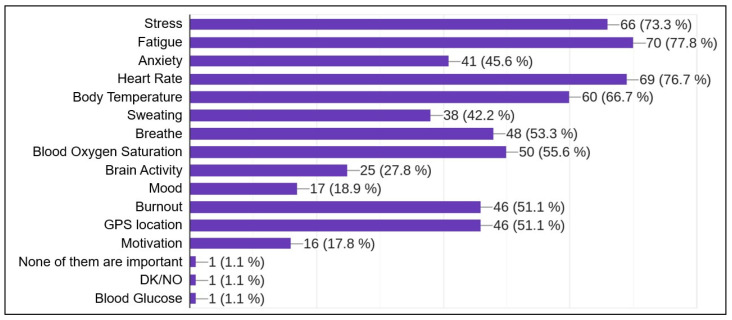
Answers to question: Items to be investigated in bio-signal monitoring.

**Figure 14:**
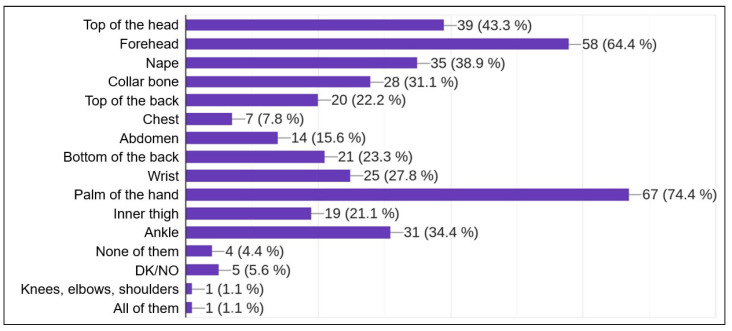
Answers to question: parts of the body where sensor placement would hinder your duty.

After an informed agreement and some personal data (age group and gender), the four main questions were:

1. Do you think that monitoring your physical and mental state during an intervention can be useful?
2. Which of the following factors do you think would be the most important to monitor while on duty? This question offers up to 15 non-exclusive possible answers (see Figure 13).
3. Would you be willing to be monitored with biological sensors during an intervention?
4. In which of the following parts of your body do you think the sensor arrangement may interfere with your work during an intervention? This question provides up to 14 possible answers, non-exclusive (see Figure 14).

This survey was sent to the Fire Brigade consortium of the Provincial Council of Málaga, which serves over 2 million inhabitants. Every question had an option for not answering (referred to as DK/NO in Figure 13 and Figure 14). A total of 95 answers were received, and 95.8% of the respondents participated in this data collection for research purposes. The gender of the participants was 95.6% male, 2.2% female and 2.2% did not answer. Regarding the age range, 7.7% were more than 55 years old, 45.1% were between 46 and 55 years, 41.8% were between 31 and 45, 4% were between 18 and 30 and, finally, 1.1% did not answer this question.

Regarding the main questions of this survey, oriented to the use of biological sensors, 97.8% of respondents agreed about the usefulness of monitoring their physical and mental state while on duty. In the question about the most interesting factors to analyze using these sensors, the most selected items were stress (72.5%), fatigue (78%), heart rate (76.9%) and body temperature (67%). A complete set of results is shown in Figure 13. Most of the firefighters surveyed were willing to be part of an experiment with biosensors while on duty (91.3%). Finally, when asked about where not to attach sensors, their main answers were the forehead (65.2%) and the palm of the hand (75%). All the results to this question can be seen in Figure 14.

This survey was composed of only a few questions to avoid disturbing firefighters’ work and revealed the interest of firefighters in biological signal monitoring in order to analyze their mental and physical conditions while on duty. Answers to the more detailed questions in the survey showed their interest in issues such as stress and fatigue, and where sensor placement would interfere with their duties: the forehead and the palm of the hand.

## 8. Wearable Sensor Suite Design

The diversity of scenarios of first responders reveals the importance of a highly reliable system for measuring their bio-signals during an intervention. The position of devices or the grasp of electrodes must not interfere with the work of responders or preclude bio-signal measurements.

**Figure 15:**
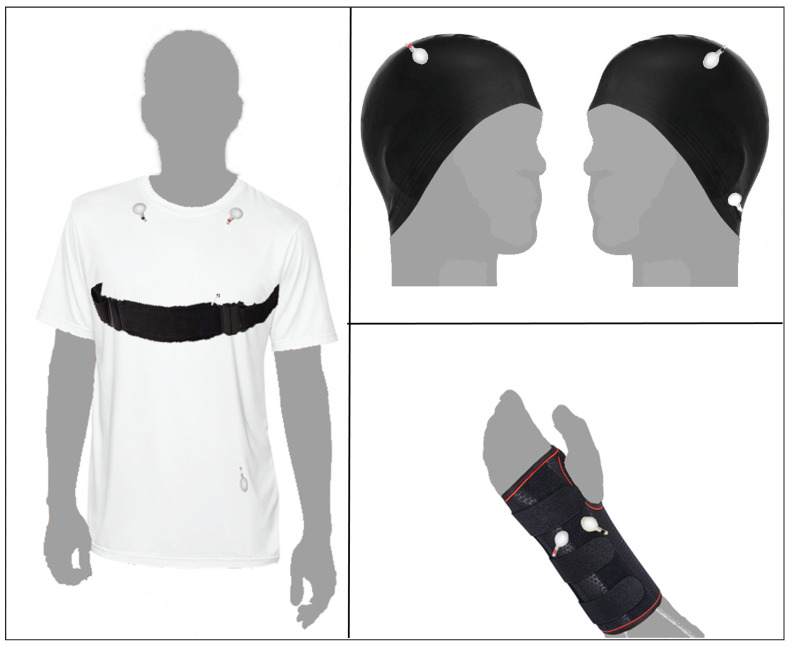
Proposal of integrated-sensor garment. Clockwise, starting from left: T-shirt with ECG and PZT sensors, integrated-EEG-sensor cap and wristband with integrated EDA sensors.

The need of decreasing the time to place sensors and improving their adherence can be solved with the development of adjustable integrated-sensor garments, which take into account ergonomics [52]. According to our experience with the exercises and the answers to the survey, a proposal of three prototypes is shown in Figure 15:

- A T-shirt with integrated ECG electrodes and PZT chest-band.
- A wristband with integrated EDA electrodes.
- A cap with integrated EEG electrodes.

The T-shirt with a fit design similar to the one currently used by firefighters integrates ECG electrodes in the position presented in Section 4, which is appropriate for fatigue and stress detection, heart rate information through an ECG signal and the PZT chest-band to monitor fatigue. The EDA signal is monitored in the wrist instead of face and palms, taking into account answers from firefighters in Section 7. This setup is not the best for stress detection but suitable according to [53]. The design of the wristband allows wearing gloves and a jacket. Finally, a cap with integrated EEG electrodes is also proposed. The placement of electrodes on the top of the head reflects concerns of firefighters for helmet comfort, avoiding the former position on the forehead. It also integrates the reference electrode behind the ear. Although mental state monitoring was not among the most selected items to be investigated during an intervention, the cap prototype has also been included to anticipate possible requirements from psychology experts.

## 9. Conclusions

This work presents a validation of remote first responder bio-signal monitoring in high-fidelity SAR scenarios while on duty. To the best of our knowledge, this is the first time that proof-of-concept experiments in disaster situations have been carried out. Furthermore, two different strategies were implemented: (1) real-time monitoring and (2) local recording of bio-signal data. For these experiments, we previously selected a sensor suite for monitoring ECG, EDA, PZT and EEG data, a key combination of bio-signals for physical fatigue, anxiety and stress detection. This set of sensors was integrated into the SAR-IoCA architecture and proven in realistic SAR missions.

With the lessons learned from these experiments, a survey was conducted among the Fire Brigade consortium of the Provincial Council of Málaga to learn whether they consider bio-signal monitoring while on duty important and their willingness to participate in future exercises. The main questions in the survey were addressed to collect their opinion about the most interesting issue to be investigated in health monitoring and in which part of their body they think that sensor arrangement may interfere with their work. Answers to these questions, along with learned lessons from the exercises, provided a reference to design three prototypes of integrated sensor garments: (1) a T-shirt, (2) a wristband and (3) a cap. Moreover, data collected from the survey and the bio-signal monitoring and recording are stored in a restricted access folder.

In the future, we plan to continue validation of the proposed wearable sensor suite design through new experiments and recording more biosensor data to accumulate a massive dataset in realistic SAR exercises. Finally, issues such as coexistence with other networks, energy consumption, and data integrity will be considered in future works.

## Data Availability

The data presented in this study are available on request from the corresponding author.
